# Coupling of Mn_2_O_3_ with Heteroatom-Doped Reduced Graphene Oxide Aerogels with Improved Electrochemical Performances for Sodium-Ion Batteries

**DOI:** 10.3390/nano13040732

**Published:** 2023-02-15

**Authors:** Nor Fazila Mahamad Yusoff, Nurul Hayati Idris, Muhamad Faiz Md Din, Siti Rohana Majid, Noor Aniza Harun, Lukman Noerochim

**Affiliations:** 1 Energy Storage Research Group, Faculty of Ocean Engineering Technology and Informatics, Universiti Malaysia Terengganu, Kuala Nerus 21300, Terengganu, Malaysia; 2 Department of Electrical and Electronic Engineering, Faculty of Engineering, National Defence University of Malaysia, Kem Sungai Besi, Kuala Lumpur 57000, Malaysia; 3 Center for Ionics University of Malaya, Department of Physics, Faculty of Science, University of Malaya, Kuala Lumpur 50603, Malaysia; 4 Advance Nano Materials (ANOMA) Research Group, Faculty of Science and Marine Environment, Universiti Malaysia Terengganu, Kuala Nerus 21300, Terengganu, Malaysia; 5 Department of Materials and Metallurgical Engineering, Institut Teknologi Sepuluh Nopember, Surabaya 60111, Indonesia

**Keywords:** sodium–ion batteries, anode, Mn_2_O_3_, heteroatom-doped rGO aerogels, electrochemical properties

## Abstract

Currently, efforts to address the energy needs of large-scale power applications have expedited the development of sodium–ion (Na–ion) batteries. Transition-metal oxides, including Mn_2_O_3_, are promising for low-cost, eco-friendly energy storage/conversion. Due to its high theoretical capacity, Mn_2_O_3_ is worth exploring as an anode material for Na-ion batteries; however, its actual application is constrained by low electrical conductivity and capacity fading. Herein, we attempt to overcome the problems related to Mn_2_O_3_ with heteroatom-doped reduced graphene oxide (rGO) aerogels synthesised via the hydrothermal method with a subsequent freeze-drying process. The cubic Mn_2_O_3_ particles with an average size of 0.5–1.5 µm are distributed to both sides of heteroatom-doped rGO aerogels layers. Results indicate that heteroatom-doped rGO aerogels may serve as an efficient ion transport channel for electrolyte ion transport in Mn_2_O_3_. After 100 cycles, the electrodes retained their capacities of 242, 325, and 277 mAh g^−1^, for Mn_2_O_3_/rGO, Mn_2_O_3_/nitrogen-rGO, and Mn_2_O_3_/nitrogen, sulphur-rGO aerogels, respectively. Doping Mn_2_O_3_ with heteroatom-doped rGO aerogels increased its electrical conductivity and buffered volume change during charge/discharge, resulting in high capacity and stable cycling performance. The synergistic effects of heteroatom doping and the three-dimensional porous structure network of rGO aerogels are responsible for their excellent electrochemical performances.

## 1. Introduction

Sodium-ion (Na-ion) batteries have good prospects as a replacement for lithium-ion (Li-ion) batteries used in electronic devices due to their low cost, vast quantity of sodium resources, and high energy density batteries, which are comparable to those of Li-ion energy storage devices [1,2,3]. Na-ion energy storage device deployment remains hampered by issues with rate performance, Coulombic efficiency, and cycle stability, among other factors. The main reason for this is that Na^+^ has a larger radius (0.102 nm) than Li^+^ (0.076 nm), resulting in slower reaction kinetics for the sodiation/desodiation of Na^+^ [4]. Ideally, anode materials should have microscopic internal structures that can store Na^+^ and enable a reversible process for sodiation/desodiation during charge/discharge. Additionally, they should be inexpensive, simple to prepare, and compatible with electrolytes. As a result, the primary challenge for Na-ion battery devices is to combine excellent anode materials with optimal electrolytes.

Because of their abundant source and high energy density, transition-metal oxides are promising anode materials for Na–ion batteries [5,6,7]. Due to their high theoretical capacity, low cost, and eco-friendliness, manganese oxides have garnered a great deal of attention [8,9]. Unfortunately, like other transition-metal oxides, their low conductivity and severe volume expansion during the charge/discharge process lead to poor electrochemical performances [10,11], limiting their practical application despite their aforementioned benefits. Several works have been reported on MnO_2_ [12,13] and Mn_3_O_4_ [14,15]; however, their electrochemical performances as an anode for Na-ion batteries are unsatisfactory. Due to its high theoretical specific capacity (1018 mAh g^−1^), Mn_2_O_3_ is thought to be one of the most promising candidates among them. However, Mn_2_O_3_ has low reversibility, poor cyclic stability, and low Coulombic efficiency as a result of the volume pulverisation during the charge and discharge process, the disintegration of its structure, and its low conductivity, which limited its applications [11,16]. Significant efforts [17,18] have been made to date to resolve the aforementioned issues. A good strategy is to use nanostructured Mn_2_O_3_ particles where the internal void and smaller particles could provide a large electrode–electrolyte contact area while also shortening ion and electron transport distances, buffering the volume change [19,20]. Another approach is to create a nanocomposite with carbon-based materials to improve the electrical conductivity between the transition-metal oxide particles while also acting as a buffer to accommodate volume changes during cycling to ensure structure stability [6,21].

**Scheme 1:**
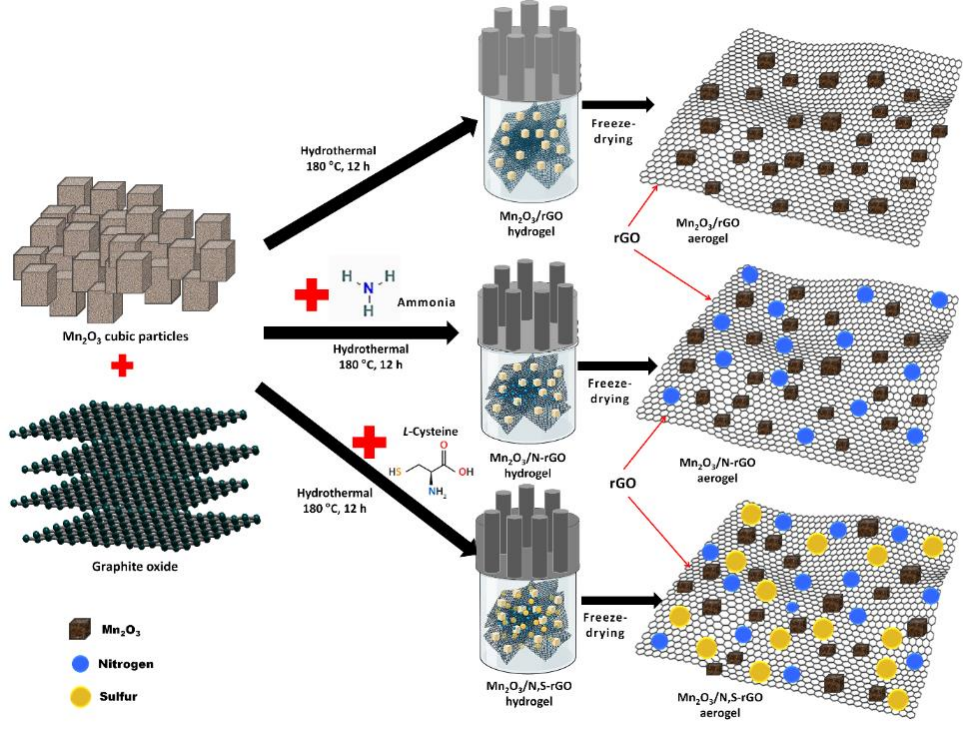
The schematic representation for the synthesis of Mn_2_O_3_/rGO aerogels and Mn_2_O_3_/heteroatom-doped rGO aerogels.

Reduced graphene oxide (rGO) aerogel has been widely used to improve the electrochemical performance of the Na–ion batteries by shortening the transportation path for Na^+^ ions through its porous aerogel structure and also to sustain the volume change [22,23]. The open channel in the porous structure of the three-dimensional (3D) rGO aerogel provides a substantial area for ion transport. This structure, which has either macropores, mesopores, or micropores, can be created simply via freeze-drying. Unfortunately, due to excessive graphene sheet stacking, graphene has a large irreversible capacity, low Coulombic efficiency, rapid capacity fading, and a low specific capacity of 372 mAh g^−1^ [24,25,26]. Doping heteroatoms such as nitrogen (N) and/or sulphur (S) could alter the physicochemical properties of rGO aerogel. It has been demonstrated that N-doping could increase the specific capacity and electronic conductivity of carbon-based materials [27,28,29,30] due to the doping defect sites and functionalized groups. S-doping, conversely, could expand the distance between graphene layers, thereby favouring the sodiation/desodiation processes [31,32,33]. The combination of these two heteroatoms, namely nitrogen and sulphur (N,S)-doping, may thus improve the electrochemical properties of Na-ion batteries through a synergistic effect. Our previous study [34] have shown the improved electrochemical performances of Mn_3_O_4_/heteroatom-doped rGO aerogel as anode in Na-ion batteries. In this work, the Mn_2_O_3_/heteroatom-doped rGO aerogel was synthesised (Scheme 1), and its electrochemical performances were investigated. The ammonia (NH_3_) and *L*-Cysteine served as the precursor of the N and N,S, respectively. During heating, the graphite oxide (GO) layers self-assembled into 3D networks due to interactions, hydrogen bonding, coordination and electrostatic interactions, while simultaneously being reduced to rGO and introducing the nitrogen and/or sulphur to the rGO layers. The heteroatom doping on the porous structure of rGO aerogel provides further assistance for Na-ion migration, and the 3D-interconnected rGO layers will provide the conductive path that is advantageous for electron transportation. Our findings may contribute to the existing body of knowledge and aid in the development of this material in the future.

The Mn_2_O_3_/heteroatom-doped rGO aerogel demonstrates an impressive discharge capacity of up to 100 cycles with remarkable Coulombic efficiencies of up to 99% in comparison with the undoped rGO. The strategy adopted here is useful in enhancing the electrode’s capacity and cycling stability. Additionally, the presence of rGO aerogel might improve conductivity and enable quick ion transport inside the electrode. The enhanced specific capacity and cyclability demonstrated by the Mn_2_O_3_/rGO and Mn_2_O_3_/heteroatom-doped rGO aerogels in our work provide new information for other researchers attempting to forecast the potential for this nanocomposite to be used in Na-ion batteries.

## 2. Materials and Methods

### 2.1. Chemicals

Graphite powders (99.99%), sulphuric acid (H_2_SO_4_), potassium permanganate (KMnO_4_) (99.0%), hydrogen peroxide (H_2_O_2_; 30%), ammonia solution (NH_3_), *L*-Cysteine, carbon black (>99.5%), polyvinylidene fluoride (PVDF), *N*-methyl-2-pyrrolidone (NMP), sodium metal (99.9% trace metal base), sodium perchlorate (NaClO_4_; 98%), propylene carbonate (PC; 99.7%), and fluoroethylene carbonate (FEC; 99%) were purchased from Sigma–Aldrich, St. Louis, MO, USA. *D*-(+)-glucose (C_6_H_12_O_6_) was obtained from Merck Millipore, and hydrochloric acid (HCl; 35.0%) was received from Alfa Aesar. All chemicals were used without further purification.

### 2.2. Synthesis of Mn_2_O_3_/rGO and Mn_2_O_3_/Heteroatom-Doped rGO Aerogels

GO was synthesised according to the modified Hummer method [35]. Mn_2_O_3_ was prepared using the hydrothermal method and then thermally decomposed, as previously reported [17]. To synthesise Mn_2_O_3_/rGO aerogel, as prepared, GO (90 mg) was dispersed in 18 mL deionised (DI) water, and then, Mn_2_O_3_ was added into the above solution. After that, the solution was transferred to a stainless-steel autoclave with a capacity of 125 mL and heated at 180 °C for 12 h. The obtained precipitates (Mn_2_O_3_/rGO hydrogel) were then freeze-dried to obtain the Mn_2_O_3_/rGO aerogel. A similar procedure was adopted to synthesise the Mn_2_O_3_/heteroatom-doped rGO aerogel, where 4 mL NH_3_ as the N source was added to the suspension and 45 mg *L*-Cysteine was added as a nitrogen/sulphur source. The collected powders were denoted as Mn_2_O_3_/N-rGO and Mn_2_O_3_/N,S-rGO aerogels.

### 2.3. Physical Characterisation

X-ray diffraction (XRD; Rigaku Miniflex II, Tokyo, Japan) was used to determine the crystal structure. The amount of Mn_2_O_3_ in the nanocomposite was quantified using a thermogravimetric analyser (TGA; Perkin Elmer, Waltham, MA, USA) at a heating rate of 10 min^−1^ in air. A scanning electron microscope (SEM; JSM-6360L; JEOL, Akishima, Tokyo, Japan) and transmission electron microscopy (TEM; TECNAI G2 20 S-TWIN FEI Company, Lincoln, NE, USA) were used to examine the morphology of the samples. Fourier transform infrared (FTIR) measurement was conducted using an IR Tracer-100 from Shimadzu (Kyoto, Japan). Raman spectroscopy (Renishaw, Gloucestershire, UK) was used to collect Raman spectra. X-ray photoelectron spectroscopy (XPS) was used to inspect the chemical states of the elements present in the composite powders using an Axis Ultra DLD XPS (Kratos, Manchester, UK), and the obtained spectra were fitted using CASA software.

### 2.4. Electrochemical Characterisation

The active materials, carbon black and PVDF (weight ratio of 75:20:5), were dissolved in NMP and then coated on a copper foil and left to dry at 100 °C overnight. The fabrication of CR2032 coin-type cells was conducted in an argon-filled glovebox (Unilab, MBRAUN, Germany, H_2_O, O_2_ < 0.1 ppm). The active material electrode was sandwiched with sodium metal (Sigma–Aldrich, 99.9%) and separated using a glass fibre (GF/D; Whatman). The electrolyte used was 1 M NaClO_4_ in PC with the addition of 5 wt.% FEC. A Neware battery analyser was used to investigate the galvanostatic charge/discharge behaviour, and cyclic voltammetry (CV) was conducted using the CHI 700E between 0.01 and 3.0 V (vs. Na^+^/Na).

## 3. Results and Discussion

**Figure 1:**
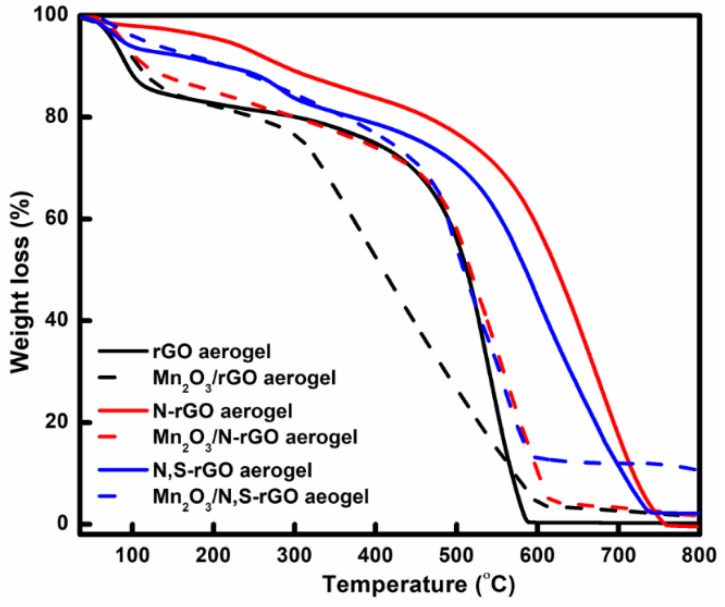
TGA curves of rGO, N-rGO, N,S-rGO, Mn_2_O_3_/rGO, Mn_2_O_3_/N-rGO, and Mn_2_O_3_/N,S-rGO aerogels.

The TGA was conducted in the air to estimate the amount of Mn_2_O_3_ in the nanocomposites (Figure 1). The weight loss from ambient until 100 °C was mainly due to the evaporation of the physisorbed water, and then the weight loss up to 400 °C occurred due to the decomposition of the hydroxyl and carboxyl groups [36] present in the rGO. Whereas, for the N-rGO and N,S-rGO aerogels, the weight loss between 150 °C and 600 °C resulted from the decomposition of nitrogen and/or sulphur-containing functional groups, respectively, and was also related to the decarboxylation and elimination of hydroxyl functionalities [37]. The rGO is completely decomposed after 600 °C [38]. Therefore, the amounts of Mn_2_O_3_ in Mn_2_O_3_/rGO, Mn_2_O_3_/N-rGO, and Mn_2_O_3_/N,S-rGO aerogels were estimated to be 15, 16, and 19 wt%, respectively.

**Figure 2:**
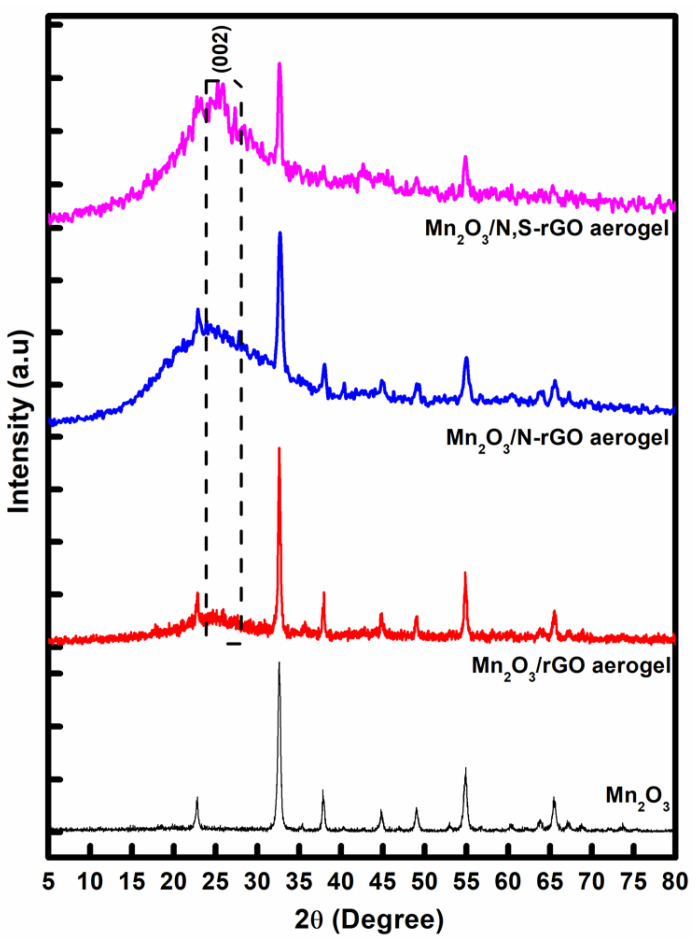
XRD patterns of Mn_2_O_3_, Mn_2_O_3_/rGO, and Mn_2_O_3_/heteroatom-doped rGO aerogels.

The XRD was used to investigate the structure of the Mn_2_O_3_ and its nanocomposites (Figure 2). No impurity phases were observed, and the Mn_2_O_3_ can be indexed to the cubic structure of Mn_2_O_3_ (JCPDS no. 11061). The presence of Mn_2_O_3_ in the nanocomposites indicates that the Mn_2_O_3_ was successfully embedded into the rGO layer structure. The broad diffraction peak appeared at 25° and was ascribed to the layered and hexagonal structure of the (002) lattice plane of the graphite-like structure [39]. The intensity of the diffraction peaks of the Mn_2_O_3_ was decreased in the Mn_2_O_3_-containing samples due to a large amount of rGO aerogel, as confirmed via the TGA.

**Figure 3:**
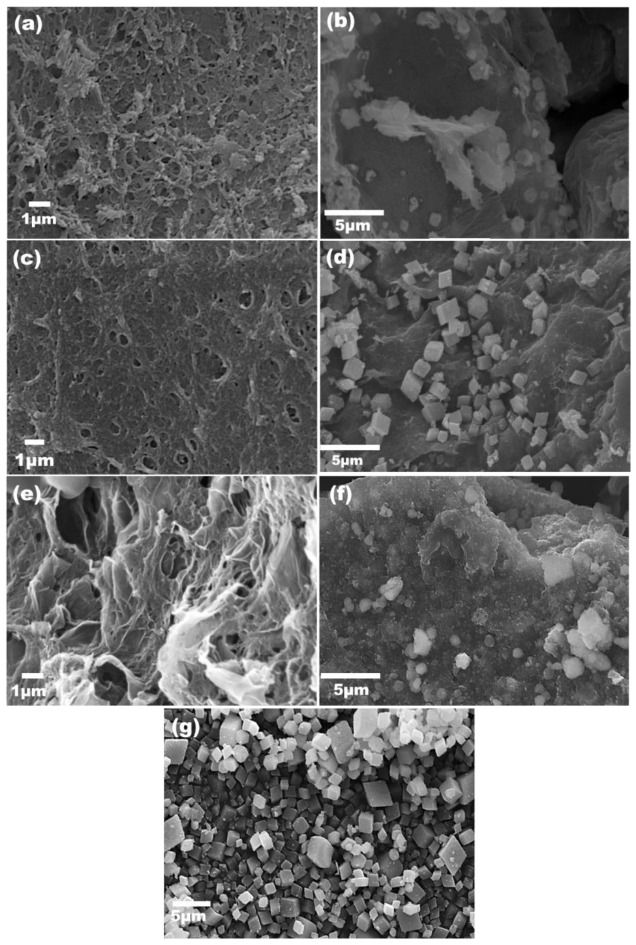
SEM images of (**a**) rGO aerogel, (**b**) Mn_2_O_3_/rGO aerogel, (**c**) N-rGO aerogel, (**d**) Mn_2_O_3_/N-rGO aerogel, (**e**) N,S-rGO aerogel, (**f**) Mn_2_O_3_/N,S-rGO aerogel, and (**g**) pristine Mn_2_O_3_.

**Figure 4:**
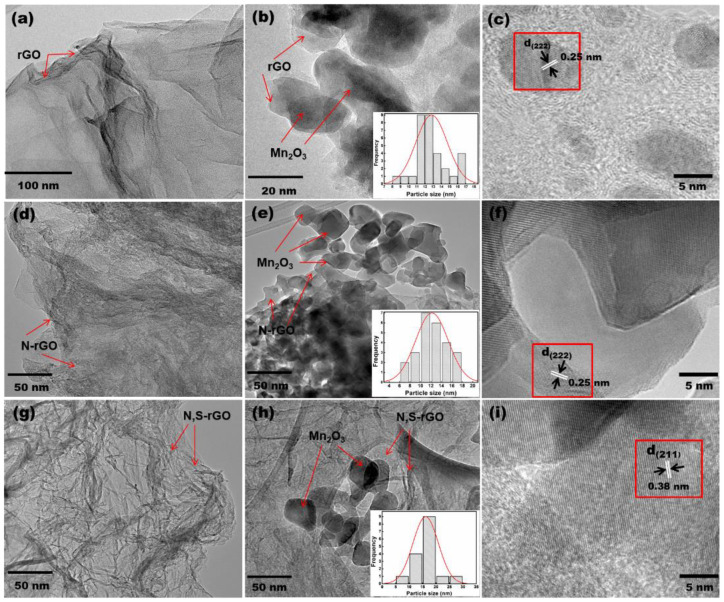
TEM images of (**a**) rGO aerogel, (**b**) Mn_2_O_3_/rGO aerogel (inset: particle size distribution), (**d**) N-rGO aerogel, (**e**) Mn_2_O_3_/N-rGO aerogel (inset: particle size distribution), (**g**) N,S-rGO aerogel, (**h**) Mn_2_O_3_/N,S-rGO aerogel (inset: particle size distribution), and high-resolution TEM images of (**c**) Mn_2_O_3_/rGO aerogel, (**f**) Mn_2_O_3_/N-rGO aerogel, and (**i**) Mn_2_O_3_/N,S-rGO aerogel.

The morphology of all samples was viewed using SEM (Figure 3) and TEM (Figure 4). Figure 3a–f shows that SEM images of the crumpled and wrinkled structure of rGO layers can be observed in all rGO aerogel-containing samples. The porous structure of rGO aerogel was interconnected through physical cross-linking to form a networked structure with pores smaller than 1 µm. The porous structure could act as an effective channel to shorten the Na–ion diffusion pathway for fast kinetics of Na–ion transport and provide a cushion to accommodate the volume changes during the charge/discharge processes [40]. Figure 3b,d,f show that the Mn_2_O_3_ cubic microstructures of 0.5–1.5 µm are distributed on the surface of the rGO layers, which are aggregates of individual Mn_2_O_3_. This suggests that Mn_2_O_3_ cubic structures and rGO aerogel layers assembled efficiently during hydrothermal. Figure 3g shows that the pristine Mn_2_O_3_ possesses a cubic morphology with a size ranging from 1.1 to 1.9 µm. Furthermore, the Mn_2_O_3_ is anchored on the surface of the heteroatom-doped rGO aerogel layers and could act as a spacer to prevent the rGO aerogel layers from restacking [41,42].

As shown in Figure 4, further investigation of the Mn_2_O_3_ anchored on the heteroatom-doped rGO aerogel layers is conducted using TEM. Typical crumpled rGO sheets formed a 3D framework (Figure 4a,d,g) aligned with the observations from the SEM images. The overlapping thin lamellar structures with clear edges and curved layers were evident in the heteroatom-doped rGO aerogels. The structural flaws caused by heteroatom doping resulted in a more wrinkled surface in the N-rGO (Figure 4d) and N,S-rGO (Figure 4g) aerogels. The TEM images (Figure 4b,e,h) further revealed the Mn_2_O_3_ were surrounded by a few rGO aerogel layers and the diameter of individual Mn_2_O_3_ is measured to be 13–17 nm based on the histogram (inset). The Mn_2_O_3_ is less transparent in comparison to its surroundings, demonstrating its porous nature. Previously, we have reported that the Mn_2_O_3_ exhibited a porous structure [17]. The high-resolution TEM (Figure 4c,f,i) demonstrated a clear interlayer d-spacing of the cubic phase of Mn_2_O_3_ of 0.25 nm in the (222) plane and 0.38 nm corresponding to the (211) plane.

**Figure 5:**
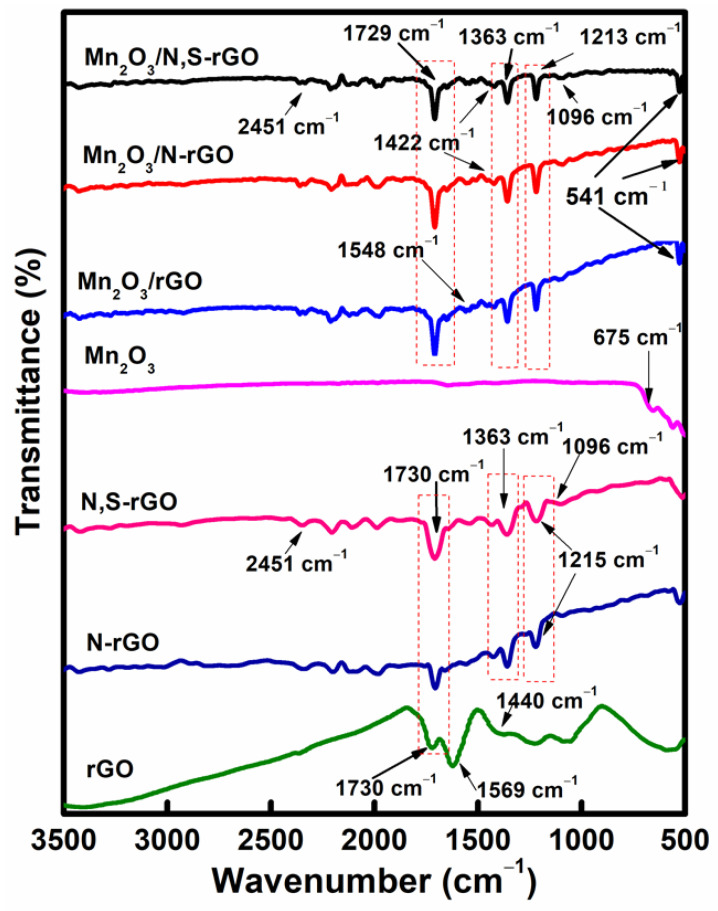
FTIR spectra of Mn_2_O_3_, rGO, N-rGO, N,S-rGO, Mn_2_O_3_/rGO, Mn_2_O_3_/N-rGO, and Mn_2_O_3_/N,S-rGO aerogels.

To support the presence of heteroatoms in the nanocomposites, Figure 5 shows the FTIR spectra of Mn_2_O_3_, Mn_2_O_3_/rGO, Mn_2_O_3_/N-rGO, and Mn_2_O_3_/N,S-rGO aerogels. The apparent peaks at 1730 cm^−1^ (C=O stretching vibration), 1363 cm^−1^ (O–H deformation), and 1215 cm^−1^ (C–O stretching vibration) indicate that GO is successfully reduced and the corresponding product is rGO [43,44]. Another FTIR peak at 1440 cm^−1^ attributed to the C–O stretching deformation and at 1569 cm^−1^ related to the aromatic skeletal *v* (C=C) stretching vibration of rGO aerogel is also observed [45]. The existence of S atoms on the N,S-rGO and Mn_2_O_3_/N,S-rGO aerogels was established via the appearance of peaks at 1096 cm^−1^ (C=S) and 2451 cm^−1^ (S–H stretching vibration) [46]. Furthermore, the C–N stretching vibration located at 1422 cm^−1^ is an indicator that nitrogen is successfully doped in the nitrogen-containing nanocomposites. The FTIR spectra of Mn_2_O_3_ also appeared in the Mn_2_O_3_/rGO, Mn_2_O_3_/N-rGO, and Mn_2_O_3_/N,S-rGO aerogels, in which the Mn–O stretching vibration appeared at 541 and 675 cm^−1^ [47]. Hence, the FTIR results indicate that there is a Mn–O, C–N, C=S and N–H bonding and thus confirmed that Mn_2_O_3_ nanoparticles were incorporated into the rGO aerogel layers.

**Figure 6:**
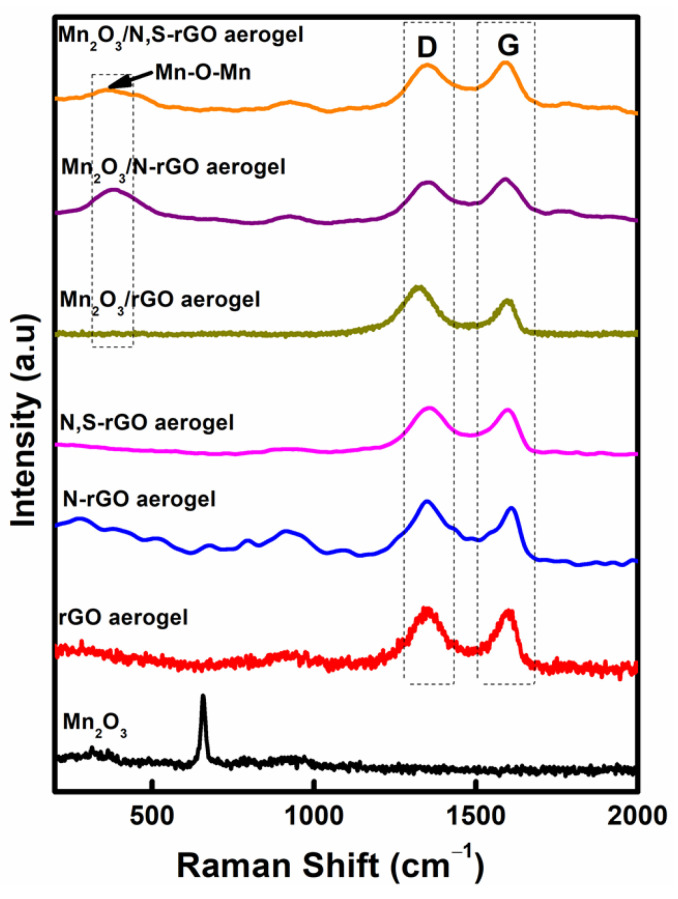
Raman spectra of Mn_2_O_3_, rGO, N-rGO, N,S-rGO, Mn_2_O_3_/rGO, Mn_2_O_3_/N-rGO, and Mn_2_O_3_/N,S-rGO aerogels.

Raman spectra for all samples are also recorded (Figure 6). Two typical characteristic peaks of carbon are observed in all samples: the D band at 1340 cm^−1^, which reflects the defect and disorder of carbon in graphitic layers, and the G band at 1590 cm^−1^, which associates with the characteristics of the E_2g_ mode of sp^2^ hybridised carbon [48,49]. The intensity ratio between the D and G bands (*I*_D_/*I*_G_) can be used to assess surface defects. According to this figure, the *I*_D_/*I*_G_ of rGO aerogel is 1.33. After nitrogen/sulphur doping, the *I*_D_/*I*_G_ of N-rGO and N,S-rGO aerogels were calculated to be 0.95 and 0.98, respectively. After nitrogen and/or sulphur doping, rGO layer defects were established, and the *I*_D_/*I*_G_ ratio was altered via the introduction of cubic Mn_2_O_3_ into graphene layers. By contrast, the calculated intensity ratios for Mn_2_O_3_/rGO, Mn_2_O_3_/N-rGO, and Mn_2_O_3_/N,S-rGO aerogels were 1.35, 1.00, and 1.02, respectively. Hence, the band located at ~340 cm^−1^ could be ascribed to the asymmetric stretch of bridge oxygen species (Mn–O–Mn), in correspondence to that obtained for bulk Mn_2_O_3_ particles [50].

**Figure 7:**
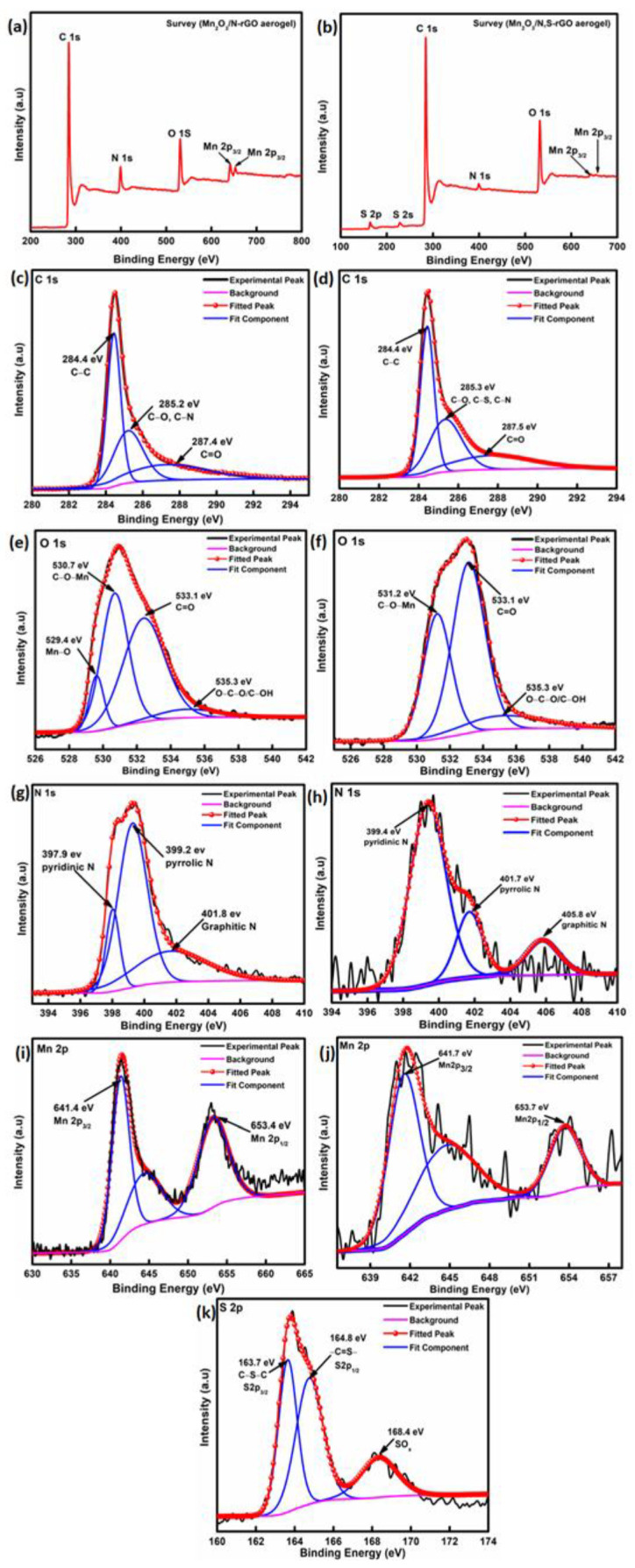
XPS spectra of the survey scan (**a**,**b**), C 1s (**c**,**d**), O 1s (**e**,**f**), N 1s (**g**,**h**), Mn 2p (**i**,**j**) for the Mn_2_O_3_/N-rGO and Mn_2_O_3_/N,S-rGO aerogels, respectively, and (**k**) S 2p spectra of the Mn_2_O_3_/N,S-rGO aerogel.

Figure 7 depicts the survey scan of the heteroatom-doped rGO aerogels and the core-level spectra of O 1s, C 1s, N 1s, S 2p, and Mn 2p for Mn_2_O_3_/N-rGO and Mn_2_O_3_/N,S-rGO aerogels. The presence of nitrogen and nitrogen–sulphur is visible in the survey spectra of Mn_2_O_3_/N-rGO and Mn_2_O_3_/N,S-rGO aerogels, respectively (Figure 7a,b). Furthermore, the O 1s, C 1s, N 1s, Mn2p_3/2_, and Mn 2p_1/2_ peaks are located at 532, 284, 399, 641, and 653 eV and are found in the survey spectrum. Figure 7b exhibits the S 2p peak located at 164 eV. Figure 7c,d shows that the C 1s region can be resolved into three peaks. The peak at 284 eV corresponds to the C–C signal, and 287 eV is related to the C=O, whereas, the peak located at 285.2 eV is related to the C–O and C–N signal for Mn_2_O_3_/N-rGO aerogel and C–O, C–N and C–S at 285.3 eV for the Mn_2_O_3_/N,S-rGO aerogel. Figure 7e,f shows the O 1s deconvoluted XPS spectra, and the peaks located at 531, 533, and 535 eV corresponded to C–O–Mn, C=O, and surface absorbed oxygen (O–C–O/C–OH), respectively. Moreover, one peak that appeared at 529 eV, as depicted in Figure 7e, might be related to Mn–O. In the N 1s spectra (Figure 7g,h), three fitted peaks can be assigned to pyridinic N (397 eV), pyrrolic N (399 eV), and graphitic N (401 eV) doped in graphene. It has also been reported that the existence of pyridinic- and pyrrolic-type nitrogens can facilitate the transfer of Na-ion and electrons, improving the rate capability of the electrodes [51,52,53]. Additionally, the N concentration in Mn_2_O_3_/N-rGO aerogel was 15.54%, whereas, for the Mn_2_O_3_/N,S-rGO aerogel, the concentration of N and S is 3.36% and 3.41%, respectively. The high-resolution Mn 2p (Figure 7i,j) can be fitted into Mn 2p_3/2_ (641 eV) and Mn 2p_1/2_ (653 eV). The observed spin-orbit splitting represented by the difference in binding energy values of these two levels is 12 eV, in line with the previous report [54]. Moreover, the S 2p can be fitted into three components of S p_3/2_, S p_1/2_ and SO_x_ centred at 163.7, 164.8 and 168.4 eV (Figure 7k), thus confirming the introduction of S atoms into disordered graphene lattice [55].

**Figure 8:**
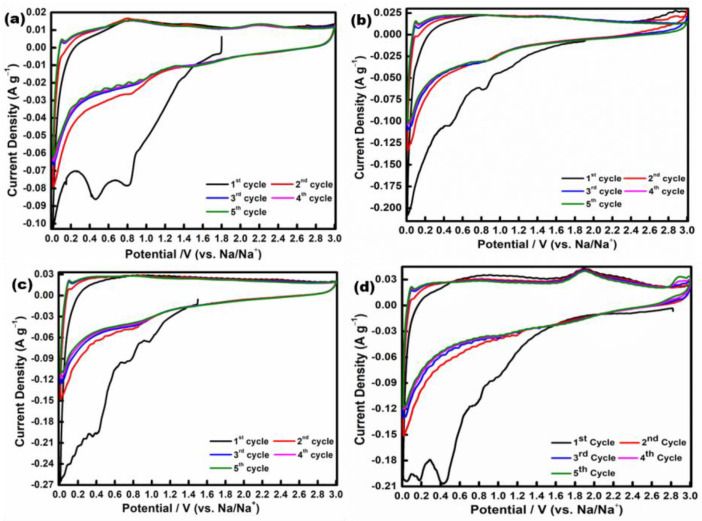
CV curves of (**a**) Mn_2_O_3_, (**b**) Mn_2_O_3_/rGO aerogel, (**c**) Mn_2_O_3_/N-rGO aerogel, and (**d**) Mn_2_O_3_/N,S-rGO aerogel at a scanning rate of 0.1 mV s^−1^.

The electrochemical activity of Mn_2_O_3_ and their nanocomposites is evaluated using CV (Figure 8) at a scan rate of 0.1 mV s^−1^ over 0.01–3.00 V. All samples show similar reduction and oxidation peaks in their CV curves. In the first cycle (Figure 8a–d), the main cathodic peak at 0.8–0.9 V attributes to the reduction of Mn_2_O_3_ to Mn−O and the formation of Na_2_O and solid electrolyte interphase (SEI) layers over the active materials, which can be expressed as Equation (1) [17,56].

Mn_2_O_3_ + 2Na^+^ + 2e^−^ → 2MnO + Na_2_O (1)

The broad peak at 0.01–0.4 V may be ascribed to the Na–ion insertion, the MnO to Mn irreversible conversion reaction (Equation (2)), and the formation of SEI on the electrode’s surface.

MnO + 2Na^+^ + 2e^−^ → Mn + Na_2_O (2)

During the second to fifth cycles, the slight shift of the reduction peak at higher potential and the overlapping CV curves indicate the electrochemical reversibility of Na-ions. Na-ion batteries exhibited wider and weaker CV peaks in comparison to Li-ion batteries since Na-ions have a larger ionic radius than Li-ion and move between graphitic carbon layers more slowly than Li-ion [57,58]. Figure 8d shows that the Mn_2_O_3_/N,S-rGO aerogel exhibited an anodic peak at 1.95 V, which may be due to the sulphur incorporated into the N,S-rGO aerogel [59]. The following reaction (Equation (3)) describes the reversible mechanism during the sodiation/desodiation

Mn_2_O_3_ + 6Na^+^ + 6e^−^ ↔ 2Mn + 3Na_2_O (3)

**Figure 9:**
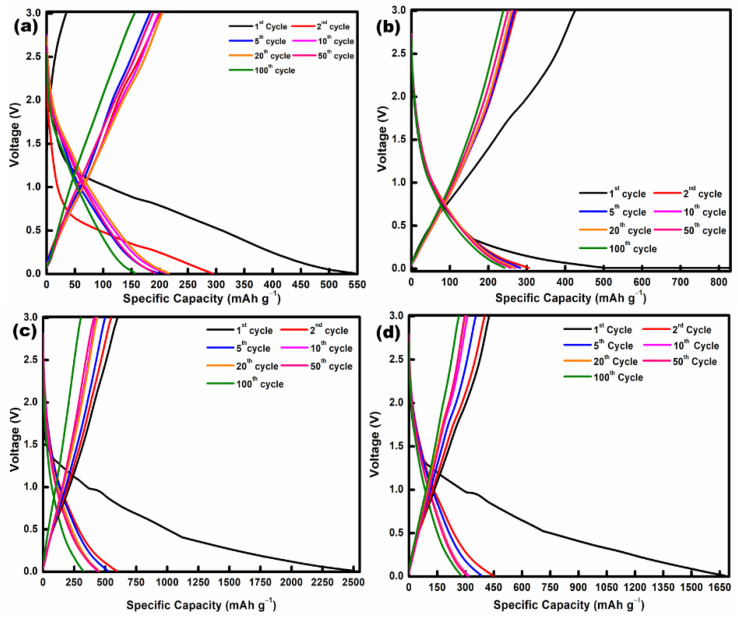
Charge and discharge profiles of (**a**) Mn_2_O_3_, (**b**) Mn_2_O_3_/rGO aerogel, (**c**) Mn_2_O_3_/N-rGO aerogel, and (**d**) Mn_2_O_3_/N,S-rGO aerogel at 0.1 A g^−1^.

Figure 9 represents the typical charge and discharge profiles of the samples for several cycles at 0.1 A g^−1^. The long discharge plateau observed at the first cycle could be due to the formation of the SEI layer, which leads to the low initial Coulombic efficiency. The subsequent cycle shows no distinct plateau, which is consistent with the CV curves.

**Figure 10:**
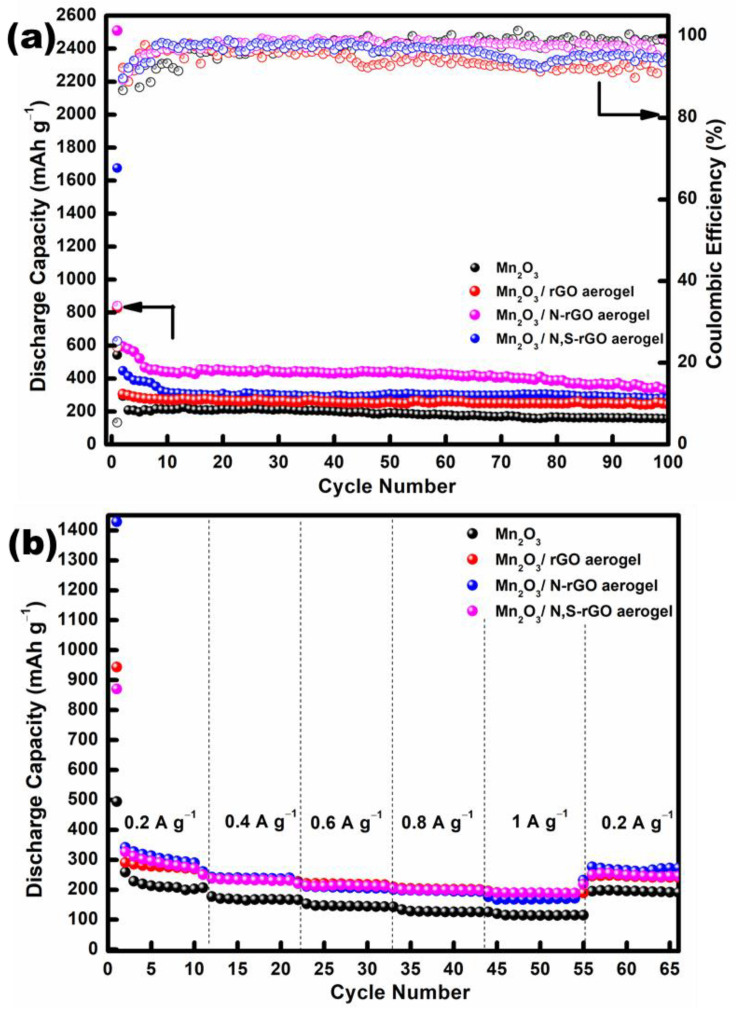
(**a**) Cyclability and Coulombic efficiencies and (**b**) rate capabilities of Mn_2_O_3_, Mn_2_O_3_/rGO, Mn_2_O_3_/N-rGO, and Mn_2_O_3_/N,S-rGO aerogels.

Further evaluation of the electrochemical performances of the Mn_2_O_3_, Mn_2_O_3_/rGO, Mn_2_O_3_/N-rGO, and Mn_2_O_3_/N,S-rGO aerogel electrodes was conducted, and Figure 10 shows the results. Figure 10a depicts that the Mn_2_O_3_/N-rGO aerogel exhibited the highest irreversible discharge capacity of 2507 mAh g^−1^, followed by Mn_2_O_3_/N,S-rGO aerogel (1677 mAh g^−1^), Mn_2_O_3_/rGO (828 mAh g^−1^), and pristine Mn_2_O_3_ (544 mAh g^−1^) at a current density of 0.1 A g^−1^. The Mn_2_O_3_/N-rGO aerogel delivers a reversible discharge capacity of 594 mAh g^−1^ during the second cycle and gradually decreases to 325 mAh g^−1^ after 100 cycles with 87% capacity retention. For Mn_2_O_3_/N,S-rGO aerogel, the reversible discharge capacity decreased from 448 mAh g^−1^ (second cycle) to 279 mAh g^−1^ (100th cycle) with an 83% retention rate. These results indicate that the heteroatom doping on the rGO aerogel layers could tailor the physicochemical properties of the rGO, which in turn enhanced the discharge capacity and cyclability of the Mn_2_O_3_. The advantages of anchoring the Mn_2_O_3_ on the rGO layers can also be seen in the electrochemical properties of Mn_2_O_3_/rGO aerogel versus pristine Mn_2_O_3_. In the second cycle, the Mn_2_O_3_/rGO aerogel exhibits a reversible discharge capacity of 305 mAh g^−1^ and gradually reaches 242 mAh g^−1^ after 100 cycles with a capacity retention of 71%. Whereas, for pristine Mn_2_O_3_, the reversible discharge capacity at the second cycle was 294 mAh g^−1^ and decreased to 157 mAh g^−1^ after 100 cycles with 71% capacity retention. During the initial few cycles, the porous structure of rGO aerogel promoted the formation of excessive SEI layers, resulting in a lower initial Coulombic efficiency [60]. After a few cycles, the Coulombic efficiencies for all electrodes reached 90% due to the relatively stable SEI that formed during the cycling. Additionally, the Mn_2_O_3_/N-rGO aerogel (Figure 10b) has better rate capability compared with other electrodes with discharge capacities of 260, 222, 201, 177, 232, and 272 mAh g^−1^ at current densities of 0.2, 0.4, 0.6, 0.8, 1.0, and returning 0.2 A g^−1^, respectively. Interestingly, other electrodes also show good rate capability performance when executed at different current densities, but with slightly lower discharge capacity values compared with Mn_2_O_3_/N-rGO aerogel.

The overall electrochemical performances of Mn_2_O_3_/heteroatom-doped rGO aerogels reported here are superior to those reported previously (Table 1). It is believed the pronounced electrochemical performances of Mn_2_O_3_/heteroatom-doped rGO aerogels can be attributed to the networked structure of the nanocomposites, which have a variety of favourable properties. First, the porous networking of Mn_2_O_3_ and rGO aerogel may shorten the diffusion path for Na-ions and facilitate the electrolyte’s penetration into the inner surface of the active materials. The interconnected rGO networks in this structure offer an additional active site for Na-ion storage and also offer a large area for reversible reactions [42,61,62]. Second, the 3D rGO aerogel networks improved the mechanical integrity of the electrode by accommodating the strain and stress during the sodiation/desodiation processes. Consequently, structure destruction of the active materials can be avoided while maintaining the cyclability of the electrode. The aggregation of Mn_2_O_3_ could also be inhibited [63,64]. Third, the electron transfer ability of the Mn_2_O_3_ was enhanced in combination with the heteroatom-doped rGO aerogel. The electrical conductivity of the active materials is greatly improved by decreasing the charge-transfer resistance, which in turn facilitates the strong electronic interconnection of the active materials to ensure rapid charge transfers within the electrode [65]. Lastly, doping nitrogen and co-doping nitrogen/sulphur improve the physicochemical characteristics of the rGO [28]. The addition of heteroatoms to rGO aerogels may improve their electrical conductivity and electrochemical activity by fostering charge transfer between adjacent carbon atoms [45,66,67]. The strategies of doping heteroatoms into rGO aerogels could be ideal platforms to tackle the problems related to the use of transition-metal oxide as anode materials for Na-ion batteries, especially electrode pulverisation during cycling. Several challenges related to Mn_2_O_3_ have to be overcome, and modifications and development of the Mn_2_O_3_ with nanocomposites are necessary to achieve the desired electrochemical properties for Na storage for practical applications.

## 4. Conclusions

In summary, the integration of Mn_2_O_3_ with the 3D framework of heteroatom-doped rGO aerogel was demonstrated to be an effective strategy to mitigate problems such as volume changes during the charge/discharge process, mechanical degradation, and capacity fading. The cubic Mn_2_O_3_ particles (with an average size of 0.5–1.5 µm) obtained via thermal decomposition of MnCO_3_ are anchored in porous and crumpled rGO sheets of micrometre dimension. This architecture ensures closed contact between the electrolyte and the active materials that enable efficient electron transport from each active Mn_2_O_3_ cubic particle to the current collector via the rGO aerogel. The aerogel structure produced heteroatom-doped rGO layers with strong interconnections, and the Mn_2_O_3_ particles distributed on the rGO layers prevented the restacking of the rGO layers. A strong synergistic effect between Mn_2_O_3_ and heteroatom-doped rGO in the nanocomposite becomes more apparent with cycling and contributes to the excellent cycling performance of the electrode. After 100 cycles, the capacities of the Mn_2_O_3_/rGO, Mn_2_O_3_/N-rGO, and Mn_2_O_3_/N,S-rGO aerogels were retained at 242, 325, and 277 mAh g^−1^, respectively. In comparison to Mn_2_O_3_/rGO, the N- and N,S-doped rGO aerogels with Mn_2_O_3_ displayed higher specific capacities, excellent cyclability, and rate capability. Thus, Mn_2_O_3_ anchored on the heteroatom-doped rGO aerogel could effectively improve the Na storage capacity of Mn_2_O_3_ and provide a useful strategy for producing high-yield anode materials for practical Na-ion batteries.

## Figures and Tables

**Table 1:** Comparison of the electrochemical performances of the Mn-based oxide anode materials for Na-ion batteries.

Mn-Based Anode Materials	Initial Discharge Capacity (mAh g^−1^)	Discharge Capacity at 2nd Cycle (mAh g^−1^)	Discharge Capacity after 100 Cycles (mAh g^−1^)	Reference
MnO/graphene	553	316	191	[68]
MnO/C	560	-	260	[69]
Mn_3_O_4_/pGS	700	-	233	[56]
Mn_3_O_4_/NC	710.8	-	249.5	[70]
Mn_3_O_4_/N-rGO aerogel	1950	470	374	[35]
Mn_3_O_4_/N,S-rGO aerogel	884	425	281	[35]
Mn_2_O_3_/N-rGO aerogel	2507	594	325	This work
Mn_2_O_3_/N,S-rGO aerogel	1677	448	279	This work

## Data Availability

Not applicable.
